# Use of Lower Limb Exoskeletons as an Assessment Tool for Human Motor Performance: A Systematic Review

**DOI:** 10.3390/s23063032

**Published:** 2023-03-10

**Authors:** Tobias Moeller, Felix Moehler, Janina Krell-Roesch, Miha Dežman, Charlotte Marquardt, Tamim Asfour, Thorsten Stein, Alexander Woll

**Affiliations:** 1 Institute of Sports and Sports Science, Karlsruhe Institute of Technology, 76131 Karlsruhe, Germany; 2 Institute for Anthropomatics and Robotics, High Performance Humanoid Technologies (H2T), Karlsruhe Institute of Technology, 76131 Karlsruhe, Germany

**Keywords:** wearable robotics, wearable devices, test, diagnosis, range of motion, strength, gait, proprioception, stiffness

## Abstract

Exoskeletons are a promising tool to support individuals with a decreased level of motor performance. Due to their built-in sensors, exoskeletons offer the possibility of continuously recording and assessing user data, for example, related to motor performance. The aim of this article is to provide an overview of studies that rely on using exoskeletons to measure motor performance. Therefore, we conducted a systematic literature review, following the PRISMA Statement guidelines. A total of 49 studies using lower limb exoskeletons for the assessment of human motor performance were included. Of these, 19 studies were validity studies, and six were reliability studies. We found 33 different exoskeletons; seven can be considered stationary, and 26 were mobile exoskeletons. The majority of the studies measured parameters such as range of motion, muscle strength, gait parameters, spasticity, and proprioception. We conclude that exoskeletons can be used to measure a wide range of motor performance parameters through built-in sensors, and seem to be more objective and specific than manual test procedures. However, since these parameters are usually estimated from built-in sensor data, the quality and specificity of an exoskeleton to assess certain motor performance parameters must be examined before an exoskeleton can be used, for example, in a research or clinical setting.

## 1. Introduction

A higher level of motor performance is associated with an increased functional performance, for example, the ability to carry out activities of daily living (ADL), as well as reduced injury and mortality [1]. With increasing age or due to injuries or illnesses, motor performance including, but not limited to, strength, balance, flexibility, endurance, or gait performance, decreases gradually or suddenly [2,3,4,5,6,7]. As a result, affected individuals may require assistive devices to carry out ADLs and remain independent. About 1.5 percent (3.6 million) of U.S. citizens use a wheelchair to maintain their mobility, and an additional 4.8 percent (11.6 million) use canes, crutches, or walkers [8]. It is estimated that about one percent of all persons across the globe use a wheelchair [9]. Research shows that motor limitations put individuals at higher risk for poverty, lower educational attainment, and lower economic and social participation, and may lead to an overall decreased quality of life [10].

**Figure 1:**
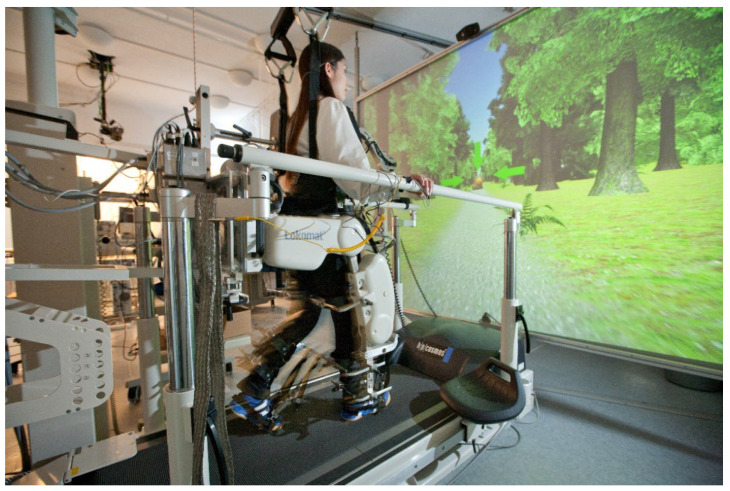
Lokomat exoskeleton. Source: Prof. R. Riener, ETH Zürich.

To overcome these problems, exoskeletons (e.g., see Figure 1) can be used to reintegrate persons with motor disabilities into social life [11]. Furthermore, they can be used to support patients as well as therapists or trainers in the rehabilitation or training progress [11,12]. Exoskeletons are mostly electrically powered robotic devices that fit tightly against the body and use rigid structures to empower or augment the user’s movements [13]. Different classifications are used for exoskeletons. The most common distinction is based on the field of application (e.g., military, occupational, medical), and the body area supported (e.g., upper limbs, trunk, lower limbs, whole body). Moreover, they can also be classified by their design, that is, anthropomorphic (some or all rotation axes of both robot and human are in alignment, and the exoskeleton replicated most of the human’s degrees of freedom (DoF)); quasi-anthropomorphic (rotation axes are not in alignment with the human joint, but the function of the exoskeleton joint is similar to its human counterpart); and non-anthropomorphic (the exoskeleton joints are in not in alignment with the human joint at all) [14]. In this review, we only considered exoskeletons for the lower limbs (whole leg or single joints), and differed stationary or mobile exoskeletons.

For control purpose, exoskeletons are usually equipped with three different types of sensors: (1) sensors to primarily detect the internal state of the exoskeleton (e.g., inertial measurement units (IMU), joint angle encoders, torque, and force sensors), (2) sensors to detect the interaction between exoskeleton with the environment (e.g., sensorized insoles or distance sensors), and (3) sensors to directly measure the state of the user (e.g., heartrate, electromyography, or electroencephalography) [15,16,17]. Sensor types (2) and (3) are used to detect motion intention of the human, and sensor type (1) is primarily used to control the movement of the exoskeletal joints, in relation to the required support of the user [18]. Due to their proximity to the body, these sensors, similar to those of other wearables (e.g., smartwatches, smart shirts, etc.), can capture and track the user’s physical and motor functions. In research and clinical practice, physical and/or motor performance data is mainly collected in stationary settings, and usually only when there is an indication, for example, during an acute illness. At the same time, data collection through clinical tests or assessments is time-consuming, costly, and personnel-intensive [19]. In contrast, novel technology, particularly portable sensors, may allow for rather easy, less-expensive, and continuous monitoring of physical and/or motor performance data of individuals [20]. According to Antonovsky’s salutogenesis model, it is assumed that the onset of a disease should not be regarded as a sudden event, but underlying pathology likely progressed and developed over a certain period of time [21]. Furthermore, health-related parameters often fluctuate even within individuals, and during periods as short as minutes or hours depending on various circumstances (e.g., stress, fatigue, weather). A prominent example of this is the “white coat effect”, which demonstrates that blood pressure rises when measured in a clinical compared to non-clinical setting [22]. Thus, a one-time snapshot measurement would not be useful/representative and sensitive enough to detect such fluctuations, or it would only detect an extreme deviation from the mean. To this end, exoskeletons, and similar wearables devices may be used to continuously record and automatically evaluate even subtle changes of certain health-related parameters [23,24,25]. A further advantage of robotic or exoskeletal assessments pertains to the collection of data that were previously not available, or were only available to a limited extent, for example, due to physical limitations of the individual [26]. With regard to motor performance, exoskeleton-derived information can be used to monitor the performance and health of the user, and to evaluate the rehabilitation progress, for example, related to gait quality (e.g., step length, step width, cadence). Furthermore, these data could inform decisions about necessary (technical) support, medical interventions, or the creation and adaptation of training plans [27].

The majority of exoskeletons already have different integrated sensors (e.g., angular encoder, IMUs, or pressure sensors). To date, these sensors are mainly used for two different purposes: first, to determine the current state of the exoskeleton, and thus to monitor and adjust forces/moments and trajectories provided by the control system; and second, to estimate the user’s movement intention, and hence, to be able to adjust the exoskeleton to the needs of the individual user [15,28].

In an initial, non-systematic review published in 2016, Maggioni et al. [29] examined the possibilities of assessing lower extremity function using robots, and regarding parameters such as range of motion (RoM), muscle strength, or proprioception. The authors conclude that future robot-supported assessment methods should not only replicate today’s established test methods, but that the advantages of the robot-supported assessments should also be used to develop new procedures. However, the ecological validity should not be compromised to ensure that such novel procedures are applicable in practice, and the data generated is useful. Ideally, the applied methods should also be transferable to other robotic devices. Overall, the authors regard the use of robots to assess lower extremity function as a “green field” that offers great potential for rehabilitation, but also requires valid and reliable methods. A second review [30] focused on the usability of rehabilitation robots to assess balance capacity. The authors conclude that robots currently have both, advantages in clinical routine (e.g., faster and more reliable examinations, testing and training with one device) as well as disadvantages (e.g., high costs and unfamiliar handling). Another review on the use of robotic technology to assess upper limb muscle strength identified five different therapy robots, and showed that these robots can reliably and sensibly quantify arm force (e.g., force magnitude, force direction, or moment of a force) [31]. Two other reviews [27,32] examined the measurement of upper limb movement performance using robots in rehabilitation with stroke patients and healthy children. The authors showed that robots can be used to record parameters such as active RoM, movement time, speed and smoothness of the motion, and precision. Furthermore, the authors note that the clinical utility of the data is not yet fully clarified, and a lack of standards makes it difficult to use and compare robots with other measurement tools. Furthermore, both reviews [27,31] mention that it is necessary to increase the validity and reliability of robot-based assessments for clinical use.

To the best of our knowledge, no review has been published that examined the use and efficacy of lower limb exoskeletons as an assessment tool for human motor performance. The specific goals of this systematic review were thus to (1) provide an overview of literature on this topic, (2) examine which parameters of motor performance (e.g., strength, joint angles, proprioception) can be/have been measured using lower limb exoskeletons, and (3) explore which approaches have been used to assess motor performance through exoskeletons. In addition, we were particularly interested in studies that compared the validity of exoskeleton-assisted measurement methods to a gold standard, as this information would be valuable to determine the validity and reliability of the data collection.

## 2. Methods

We conducted a systematic review following the criteria of the “Preferred Reporting Items for Systematic Reviews and Meta-Analyses (PRISMA)” statement [33]. This study was also registered at PROSPERO (CRD42021274215), along with a detailed description of the study protocol and methods.

### 2.1. Eligibility Criteria

The eligibility criteria for this systematic review were defined using the PICO framework [34] as follows. (1) Participants: We did not apply any exclusion criteria with regard to study population. Since exoskeletons are primarily used in therapy settings, we opted to not make any restrictions regarding diseases or physical limitations. Likewise, we did not make any restrictions regarding age of study participants. (2) Intervention: We only included studies that used lower-limb exoskeletons or wearable robotic devices for assessing motor performance. We considered all devices, regardless of whether they support only one joint (e.g., ankle, knee, hip) or multiple joints. Fixed-frame exoskeletons were also considered. Studies that examined exoskeletons for other body parts such as back, arms, or hands were not considered for this review. Similarly, rigid robots that do not have an exoskeletal structure were excluded from this review. In terms of motor performance, a wide range of parameters was considered. We were mainly interested in balance (i.e., the ability of keeping the body in a stable position thereby avoiding falls), joint angles (i.e., the angle between two body segments connected by a joint), muscle strength (i.e., the ability to generate force), proprioception (i.e., the awareness of the position and/or movement of body parts), muscle stiffness (i.e., the resistance of muscle to length change, mainly static), muscle impedance (relation between force and length of a muscle relying on a combination of stiffness, damping and mass), and various gait parameters such as phase, speed, or step length. Studies in which cognitive parameters were measured were excluded. Studies that merely simulated an exoskeleton were also excluded. (3) Comparison and outcome: No inclusion or exclusion criteria were defined regarding comparison and outcome parameters. Studies comparing data collected through an exoskeleton with a (clinical) gold standard/laboratory instrument were desired. Furthermore, we only considered studies published in English or German.

### 2.2. Information Sources

PubMed, Scopus, and Web of Science databases were used to identify articles. The search was performed in July 2021. In addition, reference lists of selected studies were screened for possible matching articles. An updated search was performed in February 2023.

### 2.3. Search Strategy

For all databases we used the following systematic search term strategy: ((exoskelet*) OR (“assistive device”) OR (“wearable robot”)) AND ((“motor performance”) OR (“strength”) OR (“balance”) OR (“flexibility”) OR (“walking function”) OR (“gait pattern”) OR (“joint impedance”) OR (“range of motion”) OR (“proprioception”) OR (“fall”) OR (“speed”) OR (“endurance”)). The search was limited to the titles, abstracts, and keywords. No further restrictions were made.

### 2.4. Selection Progress

After downloading all articles, all duplicates were removed, and the retrieved publications were screened independently by two reviewers (T.M. and F.M.) for suitability based on their title. In the next step, the abstracts of all remaining studies were again screened independently by the same two reviewers. In the final stage, we read all full texts of the remaining studies, and used our pre-defined inclusion and exclusion criteria to decide whether a study would be included in the review or not. In the case of a disagreement in any of the steps described above, a third reviewer (J.K.R.) was available for mediation. The updated search in February 2023 was only performed by one reviewer (T.M.). The literature management was done using the Citavi software (Version 6.10, Swiss Academic Software GmbH, Wädenswil, Switzerland). No automation tool was used in this process.

### 2.5. Data Collection Process and Data Items

To derive necessary information from eligible studies, a standardized data extraction form was utilized. First, all included studies were described in terms of study design and population characteristics. In the next step, we collected information about specific characteristics of the use of the exoskeleton, for example, type, sensors, sensor location, type of motor performance, activity while motor performance data were collected, the sensor-derived parameters, and possibly comparison to the gold standard. Two authors (T.M. and F.M.) independently extracted these study characteristics. If there was a disagreement or discrepancy, a third author (J.K.R.) was consulted.

### 2.6. Study Risk of Bias Assessment

The quality of each validity or reliability study included in this review was assessed independently by two authors (T.M. and F.M.). To this end, we used a modified and adapted version of the National Heart, Lung, and Blood Institute (NHLBI) Quality Assessment Tool (QA) for before–after (pre-post) studies with no control group [35]. This tool can be used to rate a study’s bias concerning research question, sample characteristics, description of the test procedure and measurement methods, and analysis of the data. As items 4, 8, 9, and 12 (inclusion of all eligible participants, blinding, loss to follow-up, and group level analysis) were not relevant to the studies included in this review, we removed these items from the assessment. The maximum possible quality score was eight points. In addition, those studies included in our review that were not validity or reliability studies, used the measurement function of the exoskeletons only as a tool, and the goals and methodological designs of these studies are of limited relevance to the content of this review. Therefore, these studies (n = 22) were not examined for their quality.

## 3. Results

**Figure 2:**
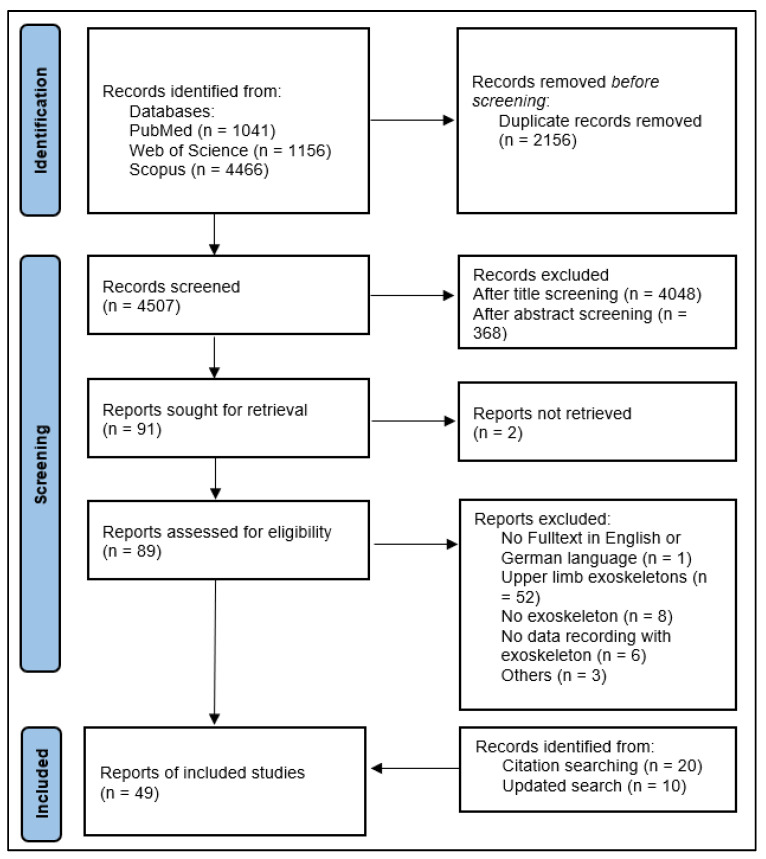
Flowchart of study section process (adapted according to [33,85]).

The initial search in July 2021 resulted in 1041 publications from PubMed, 4466 publications from Scopus, and 1156 publications from Web of Science databases. After removing duplicates, 4507 articles remained for the title and abstract screening. After both screening stages, 4461 studies were excluded, and 91 studies remained. After the eligibility screening of the full texts, another 72 articles were excluded. The main reasons for this were that the articles dealt with exoskeletons for the upper extremities, that robots were used instead of exoskeletons, and that motor performance was measured with an external measurement method instead of an exoskeleton. Furthermore, we could not retrieve the full text for two articles, and one article was written in Spanish language. Furthermore, the reference screening of all included studies resulted in 32 additional publications, of which 20 were included after the eligibility screening. The updated search resulted in 10 further studies. Thus, a final number of 49 studies were included in this review (Figure 2).

An overview of the included studies is provided in Table 1. Furthermore, in Table 2, a summary of all studies which tested the exoskeletal measurement methods for validity and/or reliability can be found.

**Table 1:** Overview and brief description of included studies (Abbreviations: 6MWT, 6-min walk test; CPR, counts per revolution; FSR, Force Sensing Resistor; IMU, Inertial Measurement Unit; n.d., not described; RS, reliability study; VS, validity study).

Author	Exoskeleton	Motor Performance Parameter	Sensors	Task	VS	RS
Joint Angles						
Lunenburger et al. [36]	Lokomat (full leg; stationary)	Hip and knee angle	Position sensors (potentiometers)	n.d.	X	X
Chaparro-Rico et al. [37]					X	X
Hu et al. [38]	Lower extremity exoskeleton (full leg; stationary)	Hip, knee and ankle angle	Encoder (hip), three degree-of-freedom (3-DOF) magnetic sensor/a pantographic exoskeleton sensor	Sit to Stand exercise	✓	X
Bryan et al. [39]	Exoskeleton emulator system (full leg; stationary)	Hip, knee and ankle angle	Magnetic rotary encoders	Walking on a treadmill	X	X
Agrawal et al. [40]	Gravity balancing orthosis (hip and knee; stationary)	Hip and knee angle	Optical joint encoders (USDigital, 2500 CPR, 1 kHz, Vancouver, WA, USA)	n.d.	X	X
Banala et al. [41]					X	X
Veneman et al. [42]	LOPES Exoskeleton (hip and knee; stationary)	Hip and knee angle	n.d.	Sagittal walking on a treadmill	✓	X
Fan and Yin [43]	Standing bed exoskeleton (hip and knee; stationary)	Hip and knee angle	Angular encoder (200 Hz)	n.d.	X	X
Koginov et al. [44]	Myosuit (hip and knee, mobile)	Thigh angle	5 IMUs (2 on each shank and thigh, 1 at the back, 100 Hz)	n.d.	✓	X
Zhang et al. [45]	Single-joint robotic hip exoskeleton (hip; mobile)	Thigh angle	IMU (50 Hz)	n.d.	X	X
Molinaro et al. [46]	Robotic hip exoskeleton (hip; mobile)	Hip angle	Absolute magnetic encoders (Orbis, Renishaw, Wotton-under-Edge, UK; 100 Hz)	n.d.	X	X
d’Elia et al. [47]	Active pelvis orthosis (APO) (hip; mobile)	Hip angle	2 absolute 17- bit Rotary Electric Encoder™ units (DS-37 + DS-25 Netzer Precision Motion Sensors Ltd., Misgav, Israel)	Walking on a treadmill	✓	X
Buesing et al. [48]	Honda Stride Management Assist (hip; mobile)	Hip angle	Angular sensors	n.d.	X	X
Pinheiro et al. [49]	Ankle–foot exoskeleton (ankle; mobile)	Ankle angle	Potentiometer (resolution: 0.5°; 100 Hz), four strain gauges, FSR (toe and heel)	Walking at 1 km/h	X	X
Bolus et al. [50]	Instrumented ankle–foot orthosis (ankle; mobile)	Ankle angle	Optical encoder (S4T, US Digital, 1 kHz, Vancouver, WA, USA), 4 IMUs (MTw Series, XSense, 50 Hz, Enschede, The Netherlands)	Walking on a treadmill at 1 m/s	X	X
Satici et al. [51]	SUkorpion AR (ankle; mobile)	Ankle angle	Angular encoder	n.d.	X	X
Park et al. [52]	Active Soft Orthotic Device (ankle; mobile)	Ankle angle	Custom-built strain sensor; IMUs	n.d.	✓	X
Aíin et al. [53]	MAFO (motorized ankle foot orthosis) (ankle; mobile)	Ankle joint angular position	n.d.	n.d.	X	X
Durandau et al. [54]	Symbitron Exoskeleton (only ankle modules) (ankle; mobile)	Ankle angle	Rotational encoder (16 b MHM, IC Haus, Bodenheim, Germany)	n.d.	X	X
Dambreville et al. [55]	Electrohydraulic robotized ankle–foot orthosis (ankle; mobile)	Sagittal plane ankle angle	Optical encoder	n.d.	X	X
**Proprioception**						
Chisholm et al. [56]	Lokomat (full leg; stationary)	Proprioception (hip and knee)	Angular encoder/potentiometer	(1) Automated movement of the joints; participants push a button when felt a movement; participant‘s legs are unloaded (2) The participant reproduces a shown/memorized position (3) The participant reproduces a memorized position	✓	✓
Domingo et al. [57]					X	X
Domingo and Lam [58]					✓	✓
Dambreville et al. [55]	Electrohydraulic robotized ankle–foot orthosis (ankle; mobile)	Proprioception (ankle)	Optical encoder, load cell	Walking on a treadmill, pushing a button if perturbation is remarked	X	✓
**Gait Phase, Spatio-temporal Gait Parameters and Walking Ability**						
Maggioni et al. [59]	Lokomat (full leg; stationary)	Walking ability (based on the required amount of support)	force sensors, potentiometers (hip and knee angles)	n.d.	X	X
Lonini et al. [60]	ReWalk (full leg; mobile)	Walking ability score (step frequency, standard deviation of the frontal angle, approximated energy expenditure, number of steps)	Accelerometer (Actigraph, ActiGraph LLC, Pensacola, FL, USA) on exoskeleton (mid-sagittal position, 20 cm above hip)	6MWT	(✓)	X
Gambon et al. [61]	EksoGT exoskeleton (full leg; mobile)	Stride time + length, gait speed + events	Resistive force sensor (heel and toe), motor encoder	Level ground walking at self-selected speed	X	X
Li et al. [62]	Unilateral rehabilitation exoskeleton robot (full leg; mobile)	Gait phase	Infrared Distance Sensors	Level ground walking at self-selected speed	✓	X
Xia et al. [63]	Passive lower limb weight-bearing exoskeleton (full leg; mobile)	Gait phase	IMU (thigh and shank, 2000 Hz)	Treadmill walking	✓	X
Kang et al. [64]	Powered hip exoskeleton (hip; mobile)	Gait phase, walking speed	Angular encoder (hip), IMU (Micro USB, Yost Lab, Portsmouth, OH, USA) (trunk + thigh)	Level ground walking at self-selected speed (between 0.8 m/s and 1.2 m/s)	✓	X
Kang et al. [65]	Gait Enhancing and Motivating System (hip; mobile)	Gait phase	FSR (only [65]), Angular encoder (hip), IMU (Micro USB, Yost Lab) (trunk + thigh); sampling rate: 100 Hz [65]; 200 Hz [66]	n.d.	X	X
Kang et al. [66]					X	X
Zhang et al. [45]	Single-joint robotic hip exoskeleton (hip; mobile)	Gait phase estimation (based on thigh angle and thigh acceleration)	IMU (50 Hz)	Different walking tasks for validation study (treadmill; level ground)	✓	X
Zhang et al. [67]	Hip joint lower limb exoskeleton (hip; mobile)	Gait phase	IMU (thigh)	Level ground walking, Stair walking (up and down)	X	X
Crea et al. [68]	Active pelvis orthosis (APO) (hip; mobile)	Gait phase	Capacitive pressure sensors	Treadmill walking at different speed	✓	X
Cao et al. [69]	Soft lower limb exoskeleton (hip; mobile)	Gait Phase	IMU (1000 Hz)	n.d.	X	X
Yu et al. [70]	Portable knee exoskeleton (knee; mobile)	Gait phase	IMUs (HI219M, HiPNUC Technology, 200 Hz)	Treadmill walking at different speed	✓	X
Pinheiro et al. [49]	Ankle–foot exoskeleton (ankle; mobile)	Gait phase	Potentiometer (resolution: 0.5°; 100 Hz), four strain gauges (resolution: 1 Nm, 100 Hz), FSR (toe and heel, 100 Hz)	Walking at 1 km/h	X	X
Bolus et al. [50]	Instrumented ankle–foot orthosis (ankle; mobile)	Gait phase	Optical encoder (S4T, US Digital, 1 kHz), 4 IMUs (MTw Series, XSense, 50 Hz), strain gauge-based reaction torque sensor (TFF350, Futek, 1 kHz), FSR (model 42, Interlink Elect., 75 Hz), pressure-sensitive capacitive films (Pedar and Pliance, Novel, 50 Hz).	Walking on a treadmill at 1 m/s	X	X
**Joint Torque and Strength**						
Galen et al. [71]	Lokomat (full leg; stationary)	Maximum voluntary isometric Hip, Knee and ankle (only [72]) torque/strength	Force transducers (integrated in every joint actuator), potentiometer	Maximal isometric contraction against the exoskeleton	✓	X
Cherni et al. [73]					✓	✓
Chaparro-Rico et al. [37]					X	X
Lunenburger et al. [36]					X	X
Tan and Dhaher [72]					X	X
Bolliger et al. [74]					X	✓
Cruz and Dhaher [75]	Motorized, instrumented exoskeletal orthosis (full leg; stationary)	Hip and knee torque	Load cells (thigh, proximal shank, and distal shank; sample rate: 1 kHz)	Maximal isometric contraction against the exoskeleton in fixed position	X	X
Agrawal et al. [40]	Gravity balancing orthosis (hip and knee; stationary)	Hip and knee torque	Optical joint encoders (USDigital, 1 kHz), two built-in force-torque sensors (ATI, 1 kHz)	n.d.	X	X
Banala et al. [41]					X	X
Fan and Yin [43]	Standing bed exoskeleton (hip and knee; stationary)	Muscular strength (isometric and isokinetic)	Angular encoder (200 Hz); Force sensor (air pressure sensor; 200 Hz)	n.d.	X	X
Rea et al. [76]	X1 exoskeleton (full leg; mobile)	Isokinetic, isotonic, and isometric muscle strength, torque, rate of change of torque,	n.d.	n.d.	(✓)	(✓)
Naghavi et al. [77]	FUM HEXA-I (hip; mobile)	Strength index for hip extension/flexion	Beam-type load-cells, 16-bit incremental angular encoder	Treadmill walking (self-selected speed)	X	X
Molinaro et al. [46]	Robotic hip exoskeleton (hip; mobile)	Hip torque	Absolute magnetic encoders (Orbis, Renishaw, UK; 100 Hz), IMU sensors (100 Hz)	Walking on the ground/ascending ramp/descending ramp	✓	X
Aíin et al. [53]	MAFO (motorized ankle foot orthosis) (ankle; mobile)	Ankle joint torque	n.d.	n.d.	X	X
Satici et al. [51]	SUkorpion AR (ankle; mobile)	Ankle torque	Angular encoder	n.d.	X	X
**Stiffness/Spasticity/Impedance**						
Riener et al. [78]	Lokomat (full leg; stationary)	Hip and knee spasticity	Force transducers (integrated in every joint actuator), potentiometer	Automated movement of the tested joints; participant‘s legs are 100% unloaded	X	X
Lunenburger et al. [36]					✓	X
Chaparro-Rico et al. [37]					X	X
Cherni et al. [79]					X	✓
Koopman et al. [80]	LOPES Exoskeleton (hip and knee; stationary)	Hip and knee impedance	Potentiometers on the exoskeleton (angles; 100 Hz) and potentiometers in the SEA (torque; 100 Hz)	Two positions (1) Hip: 5°; knee 55° (2) Hip 25° and knee 15° Two tasks: (1) Relax task (not interact with the exoskeleton) (2) Position task (position is displayed on a screen, participants should keep the error as small as possible)	X	X
Mendoza-Crespo et al. [81]	H2 robotic exoskeleton (full leg; mobile)	Ankle spasticity	Force sensors	n.d.	X	X
Nazon et al. [82]	Torque-controllable exoskeleton (knee and ankle; mobile)	Knee impedance	SSubmicron resolution optical encoders (ATOM; Renishaw, Wotton-under-Edge, Gloucestershire, UK)	n.d.	(✓)	X
Roy et al. [83]	MIT’s ankle robot system (ankle; mobile)	Ankle stiffness	Linear incremental encoders (Renishaw, Chicago, IL; resolution: 5 × 10^−6^ m), analog current sensors (Interactive Motion Technologies; resolution: 2.59 × 10^−6^ Nm);	Moving the ankle in two planes (sagittal and frontal)	X	X
Roy et al. [84]					X	X
Satici et al. [51]	SUkorpion AR (ankle; mobile)	Ankle impedance (joint angles + torques)	Angular encoder,	n.d.	X	X

### 3.1. Study Characteristics

In the 49 included studies, 33 different exoskeletons or active orthoses were examined. Of these, seven can be considered as (treadmill) stationary exoskeletons, and 26 can be considered as mobile exoskeletons. Regarding the stationary exoskeletons, four supported the full leg, whereas three supported hip and knee joints only. The most-often used exoskeleton (Lokomat, n = 12) can also be considered as “full leg” exoskeleton. None of the stationary exoskeletons examined in the included studies supported only one joint (monoarticular). As for the mobile exoskeletons, six devices supported the full leg, one device supported hip and knee joint, one device knee and ankle, and 18 devices supported only one joint (hip: n = 9; knee n = 0; and ankle: n = 9).

**Figure 3:**
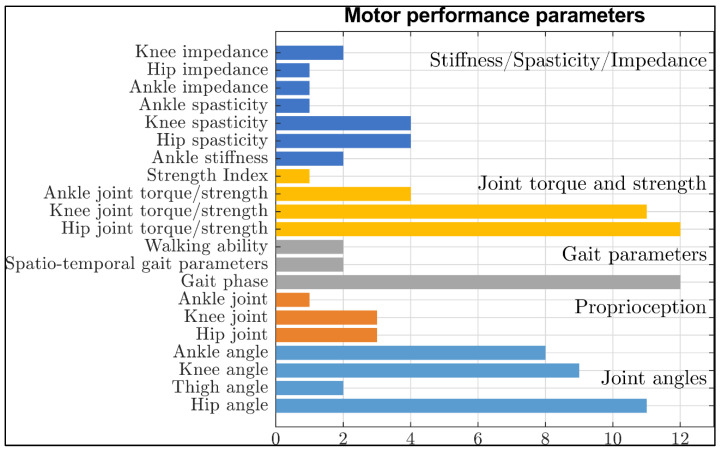
Motor performance parameters assessed in studies (please note that in some studies, multiple parameters were assessed).

**Figure 4:**
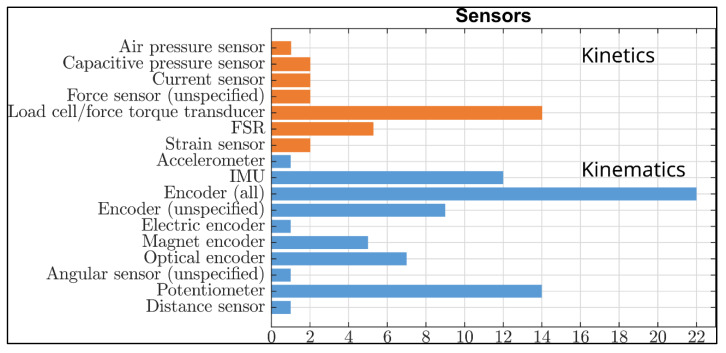
Sensors used in studies (please note that in some studies, multiple sensors were used).

The motor performance parameters measured in the studies included in this review can be categorized as follows: joint angles (n = 20), proprioception (n = 4), gait parameters or gait phase estimation (n = 14), muscle strength and joint torque (n = 15), and joint stiffness, impedance or spasticity (n = 11). Some exoskeletons were used to measure more than one parameter simultaneously. For a better overview of the measured parameters and the sensors used, see also Figure 3 and Figure 4.

Most of the studies (n = 27) used the exoskeleton as an assessment tool within a broader (intervention) study. The respective test procedure was validated in 19 studies, and tested for reliability in six studies, with three studies performing both validity and reliability testing. Two studies [51,82] only presented results from an experiment without any participants. In four studies [52,67,78,81], the number, health status, age, and sex of participants were not mentioned. The remaining 43 studies included a total of 631 participants. Of these, 48.3% (n = 305) were male, 34.1% (n = 215) were female, and sex/gender was not specified for the remaining 17.5% (n = 111) participants. More than half of all participants (58.9%; n = 372) were described as healthy, able-bodied, or unimpaired. 13.5% (n = 85) of participants had an incomplete spinal injury, and 10.1% (n = 64) had a history of stroke. Two studies [73,79] examined 33 children with cerebral palsy, and two studies [36,74] included participants with different neurological disorders. Only three studies [65,70,76] did not provide any information about the health status of their participants. The average age of participants was under 18 years in two studies, between 18 and 40 years in 25 studies, and between 40 and 60 years in three studies [71,72,75]. Only two studies [37,48] used an exoskeleton in individuals with a mean age of more than 60 years. Furthermore, three studies only provided an age range of their participants, and eight studies did not provide any information regarding participants’ age.

### 3.2. Risk of Bias in Studies

The results of the quality assessment of the validity and reliability studies are shown in Table 3. The quality of the studies ranged from 0 [76] to 8 [55,71,73,74,79] points. On average, the studies scored 5.41 points, which is indicative of good quality and low risk of bias. The main limitations were small sample sizes, and only one-time measurements of relevant parameters.

### 3.3. Results of Included Studies

In the following sections, we will provide a summary of the studies included in this review. If provided by the authors of the original research, we will present the sensors used, and the procedure which was conducted for the assessment. If the aim of the respective study was to examine validity or reliability, we will also provide a short description of the methods and results.

#### 3.3.1. Joint Angles

We included 20 studies with 18 different exoskeletons which examined the joint angles or RoM of the study participants. Of these, 6 were stationary exoskeletons, and 12 were mobile exoskeletons or actuated orthoses.

##### Stationary Exoskeletons

The Lokomat is a full leg exoskeleton, with only the hip and knee joints actuated, that measure joint angles of the user with the L-ROM (lower limb-RoM) tool [36,37]. In the studies we included, however, the sensors used for this purpose were not precisely defined, but are simply referred to as “position sensors” [86]. Since the human as well as the technical joints and segments lie on one line, the developer of the L-ROM Tool assume that the joint angles measured by the exoskeleton also correspond to the human angles [86]. The built-in tool moves the limb up to a predefined torque threshold, and then determines the maximum and minimum joint angles. Another study [38] used a three DoF magnetic sensor for sensing hip, knee, and ankle joint angle with a full body unilateral stationary exoskeleton for stroke patients. In a validation study, they showed, on average, only small (hip: 4.93° ± 3.05; knee: 3.00° ± 1.64, ankle: 1.02° ± 0.79) derivations from the measurement with an optical marker-based system (Vicon). Further, Bryan et al. [39] presented a full leg exoskeleton which is capable of measuring joint angles. They estimated that the joint angles of the exoskeleton are the same as those of the human.

In addition, the included studies used three different stationary exoskeletons which support only the knee and the hip, and are also able to assess the angles of these joints. The “Gravity-Balancing Leg Orthosis” from Banala et al. [41] and Agrawal et al. [40] is a passive exoskeleton which is mounted on a moveable frame, and can be used to assess the angles of the hip and knee joints. The developers used optical joint encoders (USDigital, 2500 counts per revolution; 1 kHz) to determine the angles of the exoskeleton, which were also aligned with the joint angles of the human being. In both studies, no explicit test was performed to determine the RoM; rather, angles during walking and leg raising were examined. The LOPES Exoskeleton [42] is a treadmill stationary exoskeleton, which can be used to determine sagittal hip and knee angles. The authors did not describe how the exoskeleton measures the angles and processes the data. However, in a validation study with 10 physically unimpaired and young participants walking on a treadmill, they observed only small differences between the robot and the human joint angles, which were recorded with marker-based motion capturing system. They conclude that errors in hip angle are thought to be primarily due to marker cluster rotations caused by muscle contractions. However, the accuracy of the measurement with the exoskeleton is not yet sufficient to calculate inverse dynamics. Furthermore, Fan and Yin [43] presented an exoskeleton which is mounted on a standing bed for early post stroke rehabilitation and evaluation of the rehabilitation progress. For angle detection, they also used an angular encoder (200 Hz). The authors did not provide any further information as to how the data were processed, or if they validated the data in comparison to another method (e.g., motion capturing or goniometer).

##### Mobile Exoskeletons or Actuated Orthoses

Regarding the mobile exoskeletons, 1 exoskeleton supporting the hip and knee, and 11 single-joint (hip (n = 4) and ankle (n = 7)) exoskeletons were used in the studies included in this review.

Koginov et al. [44] measured the thigh angle with five IMUs (100 Hz) with the Myosuit exoskeleton (supporting the hip and knee). For better results, they trained a machine learning algorithm (XGBoost). In a validation study with young and unimpaired participants who were standing and walking on a treadmill with different support and speed, they showed an average RMSE of 2.5°. Zhang et al. [45] presented a single-joint hip exoskeleton which is capable of determining the thigh angle with one IMU (50 Hz) attached along the sagittal plane of the thigh. The zero point of the thigh angle was set in a way that it was perpendicular to a plane floor. For smoothing the raw data of the sensor, a low-pass filter was used, after that they used a basic kinematic model to calculate gait parameters (see also Section 3.3.3). In this study, no explicit test was performed to determine the RoM; rather, the courses of the joint angles during treadmill walking with the exoskeleton were examined. In another study, Molinaro et al. [46] presented a hip exoskeleton to measure hip joint angles via absolute magnetic encoders (Orbis, Renishaw, UK; 100 Hz). In this work, the authors used the measured and segmented joint angles to estimate hip torques, which is presented later in this review. d’Elia et al. [47] used two absolute 17-bit Rotary Electric Encoder™ units (DS-37 and DS-25 Netzer Precision Motion Sensors Ltd., Misgav, Israel) in their active pelvis orthosis to detect hip joint angles. In a validation study, five healthy young adults walked on a treadmill under different speed and support conditions. The angles measured with the orthosis were then compared to angles measured with an optical marker-based motion capturing system. The measurement differences increased with increasing speed, and with increasing support (especially in the range between 40 and 90% of the gait cycle) by the orthosis. The root mean square error (RMSE) between the robotic angle and the human joint angle varied between 2.7° (slow speed; transparent mode) and 7.2° (fast speed; high assistance). Furthermore, Buesing et al. [48] used the Honda Stride Management Assist Device which is also capable of measuring hip joint angles with integrated angle sensors. However, no additional information was provided.

With regard to the mobile ankle exoskeletons to measure ankle joint angles, Pinheiro et al. [49] used the ankle–foot exoskeleton which has a built-in potentiometer (resolution of 0.5°; 100 Hz) as an angle position sensor. However, the authors used the raw angle data without processing or validating them. Satici et al. [51] introduced a concept to a non-anthropomorphic ankle exoskeleton called SUkorpion AR. Additionally, they describe how they derive the respective estimated joint axis from the raw data of the sensors. Another study [52] developed an “active soft orthotic device” with an artificial muscle and tendon system, and two different sensor systems (custom-built strain sensor and IMUs) to measure ankle joint angles. The strain sensors which are placed on the soft tissue in the area of the anterior ankle of the exoskeleton, must be calibrated before use with the help of the IMUs. The researchers validated the angle measurement with the strain sensors as well as with the IMUs with a commercial goniometer in only one participant. The mean error was 0.135° ± 2.85 for the IMU, and 0.255° ± 1.63 for the strain sensor while freely moving the foot for 20 min in plantar and dorsiflexion. Dambreville et al. [55] developed an electrohydraulic robotized ankle–foot orthosis which was equipped with an optical encoder to determine ankle joint angles in the sagittal plane. Also, Durandau et al. [54] used the ankle modules of the Symbitron to determine the ankle angle of the user by just reading the encoders of the exoskeleton. Furthermore, Aíin et al. [53] used a “motorized ankle foot orthosis” (MAFO) which is capable of measuring ankle joint angles, but they did not provide any information as to which sensors are used by the device, and whether the procedure is validated or not. Bolus et al. [50] presented an instrumented ankle–foot orthosis which used a large set of sensors to determine different parameters. For sensing the ankle rotation in sagittal plane, they used optical encoder (S4T, US Digital, 1 kHz), and for analyzing the orientation of the limb they integrated four IMUs (MTw Series, XSense, 75 Hz).

#### 3.3.2. Proprioception

We included four studies with two different exoskeletons which examined the proprioception of study participants. Of these, one was a stationary exoskeleton, and one was a mobile exoskeleton or actuated orthosis.

##### Stationary Exoskeletons

The Lokomat exoskeleton was used in three studies to determine proprioception or kinaesthesia. As described above, the Lokomat exoskeleton uses position sensors to determine joint angles. For proprioception, the authors [57,58] described two tasks: (1) the exoskeleton brings the joint in a predefined target position and after five seconds of rest in a second distraction position. The participants should then move their limb with a joystick back to the target position; and (2) The participants are shown the actual position of their limb and the target position with a stickman on a screen in front of them. They are then asked to move their limb to the target position with a joystick and without any visual reference. In both tasks, the difference between the target position and the achieved position was used for evaluation purposes. The first task described was tested for validity and reliability by Domingo and Lam [58] as follows: Regarding validity, the results of the robotic assessment were compared with the results of a manual clinical assessment among participants with a spinal cord injury (SCI). As a result, it was found that the clinical results correlate significantly with the results from exoskeleton-based testing. However, the authors observed a ceiling effect for the manual but not for the robotic assessment, thus leading them to conclude that the exoskeleton-based testing is more sensitive. They also observed a moderate to good test–retest reliability, with ICC ranging from 0.493 (able-bodied; hip joint) to 0.882 (SCI; knee joint).

In another validation and reliability study, Chisholm et al. [56] used the Lokomat as a tool to determine kinaesthesia. The participants (n = 34, 17 with SCI, 17 able-bodied) were strapped into the exoskeleton, and their vision to the legs was blinded. Then, their lower limbs were passively moved at different speeds. The participants were asked to push a button when they felt their limbs moving, and then indicated the direction of movement. With the help of angular encoders, the angles (hip and knee) were measured, and in the processing, a score was formed from the difference between the initial angle and the final angle. Compared to a manual assessment, the robotic assessment seems to be more sensitive, especially as the manual method shows a ceiling effect just as for proprioception. For both groups, the authors observed a very high test–retest reliability (ICC > 0.88). They further concluded that faster speeds seem to be more promising for individuals with SCI, whereas slower speeds seem to be better to determine differences in kinesthetic abilities.

##### Mobile Exoskeletons or Actuated Orthoses

We only found one study [55] which used an “electrohydraulic robotized ankle–foot orthosis” to conduct an ankle perturbation to determine the proprioceptive threshold. The participants walked on a treadmill and were asked to push a handheld button when feeling a perturbation. The system used the “Parameter Estimation by Sequential Testing” method [87] to reduce the number of tests required to identify the ankle torque disturbances threshold. The strength of the perturbation was adjusted upwards or downwards, depending on whether the participant had detected it or not. In their study on test–retest reliability among 25 participants, they observed an ICC value of 0.78, indicating a good reliability.

#### 3.3.3. Gait Phase, Spatio–Temporal Gait Parameters and Walking Ability

We included fifteen studies which examined various gait parameters such as the current gait phase or spatio–temporal parameters (e.g., stride length or cadence) of the participants. Of 14 different exoskeletons used in these studies, 1 was a stationary exoskeleton, and 13 were mobile exoskeletons or actuated orthoses.

##### Stationary Exoskeletons

We only found one preliminary study [59] which used a stationary full leg exoskeleton (Lokomat) to assess walking function. The authors successfully tested a novel control algorithm which was specifically developed for assessing walking ability based on the required amount of support. Therefore, they used force sensors and potentiometers.

##### Mobile Exoskeletons or Actuated Orthoses

Of the studies that used mobile exoskeletons or actuated orthoses to investigate gait parameters, 3 studies examined gait parameters or walking ability, and 11 studies performed gait phase estimation.

Lonini et al. [60] used the commercially available full leg exoskeleton ReWalk to investigate the walking ability of the users. To this end, the authors attached a tri-axial wearable accelerometer (Actigraph) to the exoskeleton in mid-sagittal position above the hip which recorded the data at a sampling frequency of 100 Hz. As part of the data analysis, stride frequency, standard deviation of the sagittal trunk angle, approximated energy expenditure, and number of steps were extracted from the accelerometer data to calculate a walking ability skill score with a presented algorithm. The authors tested the exoskeleton in 11 participants (6 able-bodied, 5 with SCI) with a six-minute walk test on a 30 m hallway. The study showed that able-bodied participants did not differ from those with SCI who had a lot of training experience regarding the exoskeleton. The authors also reported that the scores of novices with SCI approached those of experts after training phase. According to the authors, the score can be used to decide whether users are ready to use the exoskeleton alone at home, or need further training. In a second study [61], the authors used the full leg EksoGT exoskeleton from Ekso Bionics to investigate gait parameters such as stride time, stride length, gait speed, and gait events. To this end, they used force sensing resistor (FSR) sensors on heel and toe on each foot, and motor encoders to determine the joint angles of the exoskeleton. The authors provide information about how the different parameters were calculated, based on a 5-link planar kinematic model, but did not validate the methods. Additionally, Kang et al. [64] used a “powered hip exoskeleton” to determine walking speed. They implemented hip joint encoder, and an IMU (Micro USB, Yost Lab) on the trunk and the thigh. For data processing, the authors used a machine learning model to determine walking speed. They further used electromyography for a better model performance. However, the last aspect is not considered in this review. For validation purpose, six participants (four younger and two older adults) walked on a treadmill under two conditions (e.g., static increasing and decreasing speed, and dynamic speed). The RMSE for young participants for the walking speed under static conditions between the measured speed by the exoskeleton, and the predefined treadmill speed was 0.094 m/s ± 0.043. For the older adults walking at a slower speed, the RMSE for the walking speed was 0.061 m/s.

As mentioned above, we included 11 studies which used onboard sensors in the exoskeleton to estimate the current gait phase. Of these, two studies used full leg exoskeletons, six studies used mobile hip exoskeletons, one used a knee exoskeleton, and two used mobile ankle exoskeletons.

In one study [62], the authors used a unilateral rehabilitation exoskeleton robot to estimate gait phases/events with infrared distance sensors integrated in the shoe part of the exoskeleton. They validated their system with a marker-based motion capture system (Vicon) with 10 healthy and young participants walking at self-selected speed on a 5 m walkway and with different speeds (2/4/6 km/h) on a treadmill. After calculating the mean absolute error and processing a Bland-Altman analysis, they showed that the differences between both systems are within a mean ± 1.96 standard deviations. Another study [63] used a passive lower limb weight-bearing exoskeleton equipped with IMUs at the thigh and shank of the exoskeleton. For processing these data, investigators used a CNN-BiLSTM Network Model. To train this model, seven participants (six male, one female) walked on a treadmill at different speeds and durations (4/6/2 km/h (4/3/3 min)), 80% of these data were used for training and 20% for testing purpose. The overall accuracy was 92.99%. Compared to other models (long short-term memory (LSTM): 92.57%; gated recurrent unit (GRU): 92.39%) the authors conclude that their model has a better generalization performance and accuracy.

In two studies [65,66], the authors investigated a fully wearable hip exoskeleton called Gait Enhancing and Motivating System (GEMS). In the first study, they used a FSR sensor at the heel to detect the heel contact, as well as angular encoder and IMU sensors (sampling rate for all sensors: 100 Hz). In a later study, they only used the built-in hip angular encoder and a 6-axis IMU sensor at the trunk at 200 Hz, because they did not want to use extra sensors outside the exoskeleton. They trained and validated a neural-network to estimate the gait phases with 10 able-bodied and young participants (seven males and three females). Compared to a well-established gait phase estimation method which uses FSR sensors at heel or toe, the neural-network based method reached the same performance. In addition, Zhang et al. [45] developed a real time algorithm to estimate the gait phase, and validated this with a single-joint robotic hip exoskeleton. To this end, they used one IMU sensor with a sample rate of 50 Hz, integrated to the thigh of the exoskeleton along the sagittal plane. In a validation experiment with seven healthy and young participants, they showed that their approach has large deviations, for example, the RMSE ranged between 5.15% (treadmill walking) and 5.53% (level ground walking) compared to an FSR based approach. Also Zhang et al. [67] presented a hip exoskeleton with two IMUs at the thigh to estimate gait phases. Furthermore, Crea et al. [68] carried out a study on their active pelvis orthosis (APO). They used capacitive sensors in the cuffs between the exoskeleton and the human thigh to measure the muscle volume changes during the gait cycle, and angular encoders to measure hip flexion and extension. The data was used to calculate the current gait phase. For validation purpose, they also used sensor insoles during treadmill walking with seven healthy and young persons (four males) at three different speeds. Compared to the offline gait phase measurement with the insoles, the RMSE was 0.26 rad (transparent mode) and 0.27 rad (assistive mode). Another study [69] used integrated IMUs to determine gait phases of the user.

One study [70] used a mobile knee exoskeleton to determine gait phases with integrated IMU sensors at the thigh (200 Hz). For estimating the different gait phases, the authors trained an artificial neural network with five participants. In a validation study with three other participants, they compared their model to a ground truth measurement with foot switches and an event-based model with the same IMU data. For steady-speed walking they showed that the artificial neural network is less accurate than the event-based model in level ground walking (RMSE: 4.1% vs. 1.66%) and stair walking (RMSE: 4.58% vs. 4.55% (ascending) and 5.32% vs. 3.51% (descending)). For various-speed walking, they showed that the artificial neural network is more accurate than the event-based model on level ground walking (RMSE: 4.18% vs. 11%) and stair walking (RMSE: 6.66% vs. 15.3% (ascending) and 8.03% vs. 18.5% (descending)).

Pinheiro et al. [49] used a mobile ankle exoskeleton (ankle–foot exoskeleton) to estimate the current gait phase. The authors split the gait cycle into four phases, and used the joint data and a joint angle-based reference model to determine the current phase. For measuring the ankle joint angle, they used a potentiometer (100 Hz, resolution: 0.5°). Bolus et al. [50] examined the aforementioned instrumented ankle–foot orthosis which used a large set of sensors to determine different parameters. They integrated an FSR (model 42, Interlink Elect.; 75 Hz) to detect gait states and pressure-sensitive capacitive films to measure plantar and interface pressure (Pedar/Pliance, Novel, 50 Hz, München, Germany).

#### 3.3.4. Muscle Strength and Joint Torques

We included 15 studies which examined the produced joint torques or muscle strength of study participants. Of the nine different exoskeletons used in the studies, four were stationary exoskeletons, and five were mobile exoskeletons or actuated orthoses.

##### Stationary Exoskeletons

The Lokomat exoskeleton was used in six studies [36,37,71,72,73,74] to determine joint torques of the users with the built-in tool “L-force”. Lunenburger et al. [36] conducted a preliminary study to examine the suitability for voluntary isometric force measurement with the device. In a second study [74], the authors presented a standardized assessment for the voluntary force. To this end, they used the force transducers which are integrated in every joint actuator to measure the linear force, and the built-in potentiometer to measure the current joint angle. Participants were then fixed to the exoskeleton, and the joints were placed in predetermined positions (knee: 45° flexion, hip: 30° flexion). Then, the participants were asked to perform an extension or flexion movement against the cuff with maximal voluntary force for three seconds with a real time feedback on a screen in front of them. At the same time, the exoskeleton pushed against the human, so there was essentially no movement (isometric condition). The authors tested this procedure for inter-and intra-rater reliability with 16 young women and 14 persons with neurological movement disorders; men were excluded because of limited force capabilities of the exoskeleton. For the healthy participants, the study showed a moderate to excellent inter- (ICC: 0.72–0.97) and intra-rater (ICC: 0.71–0.9) reliability. For persons with neurological disorders, the inter- (ICC: 0.66–0.97) and intra-rater (ICC: 0.5–0.96) reliability was lower but still moderate to excellent. In a further study [71], this method was compared to a manually-assessed muscle score [88] among participants with incomplete spinal cord injury who underwent a six week robot assisted gait training intervention. The authors found a non-linear relationship between the robotic and the manual assessment. In another reliability and validity study [73], the pediatric version of the Lokomat exoskeleton was tested in 17 children with cerebral palsy. The results showed a moderate to excellent inter- (ICC: 0.8–0.93) and intra-rater (ICC: 0.7–0.93) reliability. In comparison with a hand-held dynamometer, the authors observed a strong relationship for the hip (Pearson score: r = 0.769) and knee (r = 0.609) flexors, and a moderate relation to both flexors (knee: r = 0.530; hip: r = 0.528).

In another study, Cruz and Dhaher [75] used a motorized, instrumented exoskeletal orthosis. The stationary exoskeleton covered the hip, knee, and ankle joint to determine isometric joint torques with multiple load cells (thigh, proximal shank, and distal shank; sample rate: 1 kHz) and fixed joint angles in a “toe off” (hip flexion: 10°; knee flexion: 65°) and a “mid-swing” (hip extension: 15°; knee flexion: 45°) position. Furthermore, the aforementioned (see Section 3.3.1) stationary “gravity balancing orthosis” [40,41] was also used to assess joint torques of the hip and knee joints by considering the built-in joint angular encoder and two built-in ATI 6D-force-torque sensors (thigh and shank) with a sample rate of 1 kHz. For data processing, the authors calculated joint torques using a two DoF model, and inverse dynamics. Moreover, Fan and Yin [43] examined an exoskeleton which is mounted on a standing bed for stroke rehabilitation. The device can assess isometric and isokinetic strength. For measuring interaction forces with the human body, the exoskeleton is equipped with airbags at the contact points which act as a spring-damper connection. Additionally, air pressure sensors are integrated in these airbags to measure the interaction force. In addition, joint angles were recorded with an angular encoder. Both parameters were sampled with 200 Hz. The authors did not provide any further information, for example, if they validated the data in comparison to another method (e.g., dynamometer).

##### Mobile Exoskeletons or Actuated Orthoses

Rea et al. [76] described a full leg exoskeleton (X1 exoskeleton) which is fully wearable and capable to determine isokinetic, isotonic, and isometric muscle strength, as well as torque and the rate of torque change. In comparison to a Biodex dynamometer, the exoskeleton showed a good test–retest repeatability of peak knee torque in eight participants, but no information about the test procedure, sensors, study protocol, and results were presented. In another study, Molinaro et al. [46] presented a robotic hip exoskeleton to estimate hip joint torque via absolute magnetic encoders (Orbis, Renishaw, UK), IMU sensors (sample rate: 100 Hz), and a machine learning algorithm. For ground truth and model learning, the authors used external measurement systems (force plates, motion capturing) to compute human torque via inverse dynamics in OpenSim (V 3.3.). In a validation study with five healthy participants, they tested three different models (i.e., baseline model, and two machine learning algorithms-XGBoost and a feedforward neural network), and compared them with ground truth (i.e., motion capturing and force plates and inverse dynamics in OpenSim). Regarding the RMSE for hip torque estimation, they found no significant difference between the two machine learning algorithms. Also, both methods could additionally lower the RMSE compared to the baseline model by up to 73%.

As part of the development of a new “assist-as-needed” exoskeleton controller, Naghavi et al. [77] proposed a new strength index. This index estimates the user’s overall strength of each hip separately with the help of interaction torque between the human and the exoskeleton, and position error tracking (i.e., deviation between the desired trajectory of the controller and the real position). The index has values between 0 and 1, where “0” represents a complete passive user, and “1” a user with full abilities. The authors implemented this concept in the FUM HEXA-I exoskeleton, and used beam-type load-cells and angular encoders to determine the strength index with a presented algorithm.

With the SUkorpion AR [51], which has also been mentioned above, it is also possible to estimate the ankle joint torque. For this purpose, researchers used the data of the joint angle encoder, a robust position controller and a reaction torque observer. To perform inverse dynamics calculations, the human exoskeleton system is modeled with two DoF with three universal prismatic and spherical joints (exoskeleton), and two revolute joints (user´s ankle). Furthermore, Aíin et al. [53] used a “motorized ankle foot orthosis” (MAFO) which is capable of measuring ankle joint torques, but they did not provide any information as to which sensors were used by the device, and whether the procedure is validated or not.

#### 3.3.5. Stiffness/Spasticity/Impedance

We included 10 studies which examined the muscle stiffness, spasticity or impedance of study participants. Of the six different exoskeletons used in these studies, two were stationary exoskeletons, and four were mobile exoskeletons or actuated orthoses.

##### Stationary Exoskeletons

Four studies [36,37,78,79] used the Lokomat and the integrated L-Stiff tool exoskeletons to examine knee and hip joint spasticity or stiffness.

In two conference papers [36,78], the authors presented the process of the assessment, for example, the joints (hip and knee) of the participants were moved separately and passively with different angular speeds (30°/s, 90°/s, and 120°/s). As already described above, joint angles and joint torques were automatically measured by the exoskeleton. These parameters were then used to calculate the mechanical stiffness. To get closer to the real stiffness, confounders (e.g., internal friction or gravitational effects) were subtracted. Comparing the measurement to the results derived from the Modified Ashworth Scale, which was administered to 42 patients with neurological disorders, the authors [36] observed that both values correlate with each other, but that in persons with low spasticity, the robotic assessment is less sensitive than the manual test. In another study, Cherni et al. [79] examined the inter- and intra-rater reliability of the L-Stiff tool (hip and knee flexion and extension spasticity) with a pediatric version of the Lokomat among 16 children and adolescents with cerebral palsy. The results showed a moderate to excellent intra-rater reliability for the same session (ICC: 0.69–0.95), and a moderate to good intra-rater reliability for different day assessments (ICC: 0.49–0.89). The inter-tester reliability was poor to moderate (ICC: 0.32–0.7).

Koopman et al. [80] used the LOPES exoskeleton (hip and knee support) to estimate hip and knee impedance. For measuring joint angles and joint torques, they used a potentiometer on the exoskeleton (hip and knee angle) and a potentiometer integrated in the series-elastic actuators (torque). Both potentiometers record data with a sampling rate of 100 Hz. For the calculation of individual parameters, the authors used a simplified kinematic leg model, which represents two pendulums (leg and exoskeleton) coupled with a parallel spring damper. For testing, the protocol had two different positions (1: hip: 5°; knee 55°; 2: hip: 25°; and knee 15°), and the participants were asked to complete two tasks: (1) relax task (i.e., wearing but not interacting with the exoskeleton), and (2) position task (i.e., position is displayed on a screen, participants should keep the error as small as possible).

##### Mobile Exoskeletons or Actuated Orthoses

There was only one study that used the mobile, full-leg H2 robotic exoskeleton [81] to measure ankle spasticity. The authors used force sensors located in the foot sole and in the instep brace. Interaction forces were then used to determine spasticity.

One study [82] presented a novel approach to determine knee impedance during walking with a torque-controlled knee exoskeleton with a passive ankle joint. To this end, the investigators used a perturbation-based approach during walking. After a mechanical validation, the authors are planning to use it to estimate human knee impedance.

Furthermore, we included three studies [51,83,84] with two different exoskeletons using mobile ankle exoskeletons for measuring ankle stiffness or impedance.

The “MIT’s ankle robot system” [83,84] is an ankle robot with three DoF (only two are actuated). For estimating ankle stiffness, the investigators used linear incremental encoders (Renishaw, Chicago, IL), and analogue current sensors (Interactive Motion Technologies) for detecting ankle torque and joint angle displacement, and calculated the ratio of them. For measuring passive ankle stiffness, the ankle was moved by the robot with a constant speed (5°/s), and during testing, the participants were asked to not move their ankle. To calculate joint angles, torques, and stiffness, the authors used a rather simple linearized mathematical model based on the shank–ankle–foot system, anthropomorphic data, and geometry. In addition, Satici et al. [51] used the aforementioned SUkorpion AR exoskeleton to calculate ankle impedance through joint angle and joint torque.

## 4. Discussion

The aim of this review was to (1) provide an overview of studies and devices which used or tested lower limb exoskeletons to assess motor performance, (2) examine which parameters of motor performance can be or have been measured by lower limb exoskeletons, and (3) explore which approaches have been used to assess motor performance through lower limb exoskeletons.

### 4.1. Studies and Devices Which Used or Tested Lower Limb Exoskeletons to Asses Motor Performance

Regarding our first research question, we were able to identify a variety of different exoskeletons with built-in sensors that can be used to measure motor performance. However, there was large heterogeneity across studies regarding the design of the exoskeletons’ structure. Most studies used the treadmill stationary “Lokomat” exoskeleton, which was developed primarily for therapy purposes. Indeed, stationary exoskeletons have important advantages regarding training and testing. For example, the user does not need to carry the weight of the exoskeleton, and their own weight can, in turn, be carried by the device. Furthermore, larger sensors can be used, and loaded parts can be designed stronger. This is important, especially regarding force measurement. Furthermore, the laboratory-like conditions allow for standardized assessments to be carried out; however, the device can only be used at its current location. Mobile exoskeletons can be used more flexibly and in different environments including ambulatory settings, which may be more pertinent to the living conditions of most individuals. Here, monoarticulated exoskeletons appear most effective due to their lower complexity. Only four of the exoskeletons included in our review are freely wearable, and support the entire leg at the same time. However, it is more difficult to perform a standardized or even continuous assessment without knowing the confounding variables (e.g., surface conditions) that inevitably occur in the field. There are also differences in the replication of the joints, as most exoskeletons are (quasi-) anthropomorphic and match the number and position (same axis of rotation) of joints of the human body (not necessarily the DoF). Only few exoskeletons, such as the one presented by Satici et al. [51], are not anthropomorphic. An anthropomorphic exoskeleton has the advantage of being able to estimate different parameters with rather few sensors, which in turn reduces complexity. Non-anthropomorphic exoskeletons, where one human joint is represented by multiple joints in the exoskeletons, require multiple sensors and algorithms for calculating the values are more complex and also more error-prone. These types of exoskeletons have the possibility to reduce joint misalignments. Regarding the data processing from the acquired sensor data, some studies used unprocessed raw data to estimate human parameters, or did not specify which algorithms were used to process the data. The question as to how valid and accurate these estimates were, was only addressed in a few studies. For example, in one study [47], the results varied by up to 7° from ground truth for RoM. However, of note, the results depend on many variables such as support level and movement speed. When the calculation method was provided, mostly the simplest possible kinematic models were used. Only few studies used somewhat more complex multibody models, such as shown in Koopman et al. [80]

### 4.2. Parameters of Motor Performance, That Can Be or Have Been Measured by Lower Limb Exoskeletons

Regarding our second research question, we were able to show that a variety of motor parameters can be measured with exoskeletons (e.g., joint angles; proprioception; gait parameters such as gait phase, spatio–temporal gait parameters, and walking ability; muscle strength and joint torques; and stiffness, spasticity, and impedance), which will briefly be discussed in the following sections. These parameters represent a broad range of motor performance variables, which are particularly important for rehabilitation settings, or may be used to identify the success of a certain therapy or practice schedule. However, our review could not identify studies that use exoskeletons to assess other areas of motor performance such as dynamic balance, fall prediction, metabolic costs, or endurance which are equally important regarding training of older adults or individuals with limited motor skills. Nevertheless, balance and fall prediction can be evaluated with wearable sensors such as IMUs, and the results may also be transferable to exoskeletons [89,90]. Some exoskeletons already are being prepared or systems for exoskeletons are being developed to measure stability for control purposes [91,92]. Thus, more research is needed that uses exoskeletons to assess a broad range of motor performance variables, including balance which is associated with risk of falling.

### 4.3. Approaches to Assess Motor Performance through Lower Limb Exoskeletons

Regarding our third research question, various aspects are important to mention. First, defining the goal of an exoskeleton-supported assessment is essential for choosing the right approach. Such goals may include, but are not limited to, a clinical evaluation, control, or assessment of a training progress, the examination of motor parameters during the daily training cycle, or the examination of specific motor conditions (e.g., recognition of gait patterns). Each goal has its own requirements with regard to the assessment, the design of the exoskeleton, and the selected sensors [16]. However, in general, it is recommended to validate all parameters against a gold standard, regardless of whether they are clinically relevant or represent a newly developed exoskeleton-based parameter. In addition, if inverse kinematics or dynamics are to be calculated using a model of the musculoskeletal system, the values measured by the exoskeleton must have the same accuracy as measurement devices in the laboratory. Based on current models in prior research [42], the data was not (yet) of sufficient quality, or was not tested for such biomechanical model calculations. Almost all measurements are estimates of reality, since angles, forces, etc. of the exoskeleton are used to extrapolate to those of the user. Thus, it is particularly important that the type of sensors, their number and placement on the exoskeleton, as well as the algorithms for calculating the estimate, are optimally matched. To this end, initial studies are using machine learning to interpret selected values derived from the data; however, all studies included in this review, and using machine learning, only included healthy participants. It is thus not known how well these systems would work with individuals who are older and/or have impairments such as gait deficits. According to the authors of the included studies, this has the advantage of lower required computing power and more robust results, which are also needed for real-time control of the exoskeletons.

Compared to other testing methods, assessments of motor performance with exoskeletons have several advantages. One major advantage is the mobility of the exoskeleton. Particularly with mobile exoskeletons, assessment is possible outside of the laboratory or clinical setting. Furthermore, exoskeletons seem to be more objective and sensitive compared to manual testing procedures, particularly with regard to assessing proprioception or strength (e.g., [56]). In addition, some manual procedures and standard clinical tests have a ceiling effect, which does not appear to be the case with exoskeleton-based assessments. Moreover, exoskeletons are increasingly used in the therapy setting, and no other special equipment would then be needed to perform motor performance assessments. Most active exoskeletons generally already have built-in sensors for control, which may additionally be used for testing purpose, for example, to measure different motor parameters such as RoM in selected joints and proprioception with small additional effort. Another advantage of exoskeletons is that the data can be collected in real time, and even during training phases. Thus, even short-term effects, for example during motor adaptation [93], can be examined and better understood, as well as factors influencing current or future training sessions. Finally, individuals who cannot undergo standard test procedures, for example, due to physical limitations, may be tested using exoskeletons.

However, several limitations of exoskeletons still exist and should be addressed in future research. For example, the human–exoskeleton interface, and especially the connection points, inevitably lead to interactions that can affect the measurement of motor performance, or can alter the biomechanics of the human gait. Furthermore, if the weight of the exoskeleton is too heavy, and/or the control of the exoskeleton is poorly tuned to the human movement, the user can be forced to also carry the weight of the exoskeleton which also could lead to biases in measurement of motor performance. In addition, joint misalignments between the exoskeletons and the body cause shear forces (e.g., due to the moving center of rotation of the knee joint), what could be perceived as uncomfortable and could have a negative impact on performance. Furthermore, it can be an unfamiliar feeling for the user to wear an exoskeleton closely fitting to the body, which in turn may affect performance. During movement, the soft tissues may also move and cause further deviations in measurements. In addition, the settings of the exoskeleton in terms of control and support mode have a main impact on the measurement of motor performance [94]. The advantage of being able to test persons who were previously unable to undergo a test, also creates new challenges. For example, both the measured and the individual support provided by the exoskeleton are included in the result, and therefore the results need to be interpreted individually and carefully. Potential solutions for these limitations include a sufficiently long and standardized familiarization phase, so that the user can familiarize themselves with the new conditions (i.e., wearing an exoskeleton). The exoskeleton can only be used as a measuring instrument if and when the user in combination with the exoskeleton can constantly deliver the best possible performance. For example, Poggensee et al. [95] reported that users of their exoskeleton required 109 min of training to become an expert user, which would be needed to conduct motor assessments. For future research, this time period should be minimized by adapting the control algorithm or design of the exoskeleton. However, one can hypothesize based on the results of Poggensee et al. [95], that this familiarization or the design of the exoskeleton could also lead to an unwanted adaptation of the user to the exoskeleton, in which case the measured results would not reflect the reality without exoskeleton, but only the performance of the user while wearing the exoskeleton, which could, for example, be indicated by an alteration in stride width. In that case, the measured values would need to be interpreted cautiously and would only apply to the condition when the user is wearing an exoskeleton. Therefore, a better approach may be to reduce the potential disturbance of the measurement. This could be made possible by a special test-control algorithm, which keeps the interaction in an optimal range, or through sensor–actuator units, which always adapt the sensors to the optimum in the sense of the measurement. In any case, a reliable validation of the data is important, so that one can ideally rule out the possibility that certain disturbance variables have an influence on the measurement.

### 4.4. Motor Performance Parameters

#### 4.4.1. Joint Angles

For detection of joint angles, all exoskeletons used in the included studies measure their internal state of the exoskeleton and estimate the position of the human limb. To this end, various sensors (e.g., encoders, IMUs) are used. The minimal sample rate which should be used to track joint angles depends on the expected speed of the movement and the type of analysis to be conducted (e.g., trajectory detection or activity classification) [96]. Payton et al. [97], for example, recommend a minimal sampling rate of 25 to 50 Hz for a gait analysis, and 200 to 500 Hz for high speed sports activities like a serve in tennis. With one exception [51], all exoskeletons had a (quasi-) anthropomorphic design, which can simplify the estimation of joint angles. This is because the state of the exoskeleton (e.g., joint angles), which is often used for control reasons, can be congruent to the state of the user. Therefore, it may be sufficient to test this congruence for validity and reliability. Five validation studies were found, which showed small deviations between the estimated joint angle and the joint angle measured with the validation tool. However, the results depend on various factors such as supporting mode of the exoskeleton, and movement speed. Furthermore, the accuracy was not always high enough to perform inverse dynamics calculations. Especially in the extreme positions (maximum possible of the RoM) deviations could occur, which should also be investigated in future evaluation studies.

#### 4.4.2. Proprioception

Three studies [56,57,58] used the Lokomat exoskeleton for proprioception assessment. For position detection, they used the same angular detection sensors as mentioned in the joint angular Section 4.4.1.

In contrast to manual testing of proprioception, using an exoskeleton has the advantage that most parameters are standardized (i.e., movement speed, quantification of the response). Furthermore, the Lokomat exoskeleton is also capable of controlling the limbs with a joystick, which means that also physical impaired people can be tested. We found no study which investigated the use of an exoskeleton as an assessment tool for position reproduction for the ankle joint, which may be important for standing balance and fall prediction [98]. In addition, the influence of the robot on the proprioception is not clear, and given the contact between the robot and the exoskeleton, a measurement bias is possible.

#### 4.4.3. Gait Phase, Spatio–Temporal Gait Parameters and Walking Ability

Most studies included in this review and investigating gait parameters determined gait phases based on different sensor data (e.g., FSR sensors, accelerometers). As mentioned before by Maggioni et al. [29], exoskeletons with variable support algorithms offer the possibility to identify weaknesses in the individual gait phases more precisely, and to focus training processes on them. One main prerequisite is that the exoskeleton itself has no influence on the gait, which is possible depending on the design and the controls [99]. Only two included studies focused on other gait parameters (i.e., stride time, stride length, gait speed). Since these parameters are important for rehabilitation progress and fall risk assessment, they should be given greater focus in the future [100,101]. Two studies [59,60] proposed their own index to determine walking ability. These values calculated based on different sensor data can, for example, describe the initial state or the training progress of the user, and also identify deficits in individual motor performance sub-areas.

#### 4.4.4. Muscle strength and Joint Torques

Linear forces are often measured using force sensors (e.g., load cells, air pressure sensors) attached to the contact points between the human body and the exoskeleton, and the human forces are then calculated by using the known joint angles of the exoskeletons. A second way to determine joint torques is the implementation of a reaction torque observer (sensor). The recommended sampling rate for isokinetic measuring is 1000 Hz, but also a lower sampling rate at 500 Hz seems to provide an acceptable accuracy [102]. Not all of the studies included in this review fulfill these recommendations, and some only used sensors with a sensor rate of less than 500 Hz. Only one study [77] used the interaction forces between the exoskeleton and the user to calculate an individual strength index, which lies between 0 and 1, and is used to control the exoskeleton. However, use of the index for the evaluation of training progress is also conceivable. For the Lokomat, researchers observed a fair to good reliability, and a correlation with the results of a handheld dynamometer. Especially when compared to handheld dynamometers, exoskeletons seem to have an advantage due to higher inter-tester reliability [73].

#### 4.4.5. Spasticity/Stiffness/Impedance

The most common clinical tool to measure spasticity is the Modified Ashworth Scale, but different robotic assessment tools were also developed. However, there is no objective gold standard for the measurement of spasticity to which the results could be compared [103]. Nazon et al. [82] state that exoskeletons must be able to perturbate (apply torque), to measure the applied torque and to determine the position displacement during the perturbation to measure impedance. The authors [82] state that until now, there are no empirical biomechanical studies investigating dynamic tasks with regard to knee impedance. With regard to the Lokomat exoskeleton, the studies included in this review showed that results correlate with the Modified Ashworth score, and have a good to excellent intra-rater reliability (for children with cerebral palsy). For stroke patients, the exoskeleton was able to detect small changes after a training period, but the device cannot discriminate between lower spasticity levels. Also, other (ankle) exoskeletons seem to be feasible as a testing device for spasticity, but further validation studies are needed.

### 4.5. Limitations

Several limitations exist pertaining to the studies included in this review. First of all, we rely on a detailed and scientifically sound description of the methodological approach of each study. Unfortunately, many studies did not provide such detailed descriptions; for example, regarding the type of sensors, positions, and sampling rates used. Also, the evaluation methods (algorithms) were often not described in detail, or only described to a limited extent. Further, the target population for exoskeletons in general is very heterogeneous. Research on exoskeletons is primarily carried out in the medical field [104], in addition to military use or use in the working environment [105]. Here, the focus is on diseases that severely restrict movement, such as stroke or paraplegia. There is also an increasing focus on supporting older individuals who have restricted mobility, for example, due to age-related sarcopenia. The participants of the studies included in this review, however, were particularly young healthy men, as well as patients with neurological disorders. Future studies on exoskeletons to measure motor performance should aim at recruiting more women, older persons, and individuals with pre-existing diseases in addition to neurological conditions.

Another limitation of the included studies is the lack of validation and reliability tests. For example, for some exoskeletons it was assumed that the joint angle of the user matches that of the exoskeleton; but this may not always be the case.

There are also some limitations pertaining to this review itself, such as the identification of articles based on our predefined inclusion and/or exclusion criteria. However, we tried to overcome these limitations by carrying out the entire screening process independently by two authors. Furthermore, we conducted a reference screening of all included studies to detect more studies which ultimately led to the inclusion of 20 additional studies. Another limitation pertains to the fact, that the definition of an exoskeleton is not always clear, and has overlaps with the area of orthotics. Therefore, we always used the terminology as used by the authors of the studies included in this review. Additionally, this review only focuses on exoskeletons designed for the lower extremities. However, exoskeletons for the upper extremities may also be used to assess motor performance (e.g., strength), and results from such studies may also be transferable to exoskeletons for lower extremities.

### 4.6. Recommendations for Future Developments and Test

Although the design of exoskeletons is highly dependent on the goal of the research study or area of application, (quasi-) anthropomorphic exoskeletons reduce complexity and simplify the measurement of motor performance. Some exoskeletons may also not be suitable to conduct motor performance assessment due to design or safety features, and thus would need to be adapted. Furthermore, it is recommended to build the testing model (software architecture) in a way so that it can be used by different exoskeletons for measurement. In order to generalize the calculation of different motor parameters, one possible approach may be to create different levels in this model, and use a converter to unify the input data across devices. However, this approach would need to be validated for each device individually, for example, a current study tested their simulated model against a marker based system and found consistent results with regard to joint angles [106]. Furthermore, it has been shown that exoskeletons can also have a direct impact on motor performance (e.g., balance [107]). Therefore, further research is needed that investigates whether and how these influences can be reduced to a minimum, or how they can be adjusted. Similarly, research often focuses on reproducing quantitative or laboratory-based testing procedures using exoskeletons, or using exoskeletons to design a new procedure [108]. Less frequently, (semi) subjective methods are used, which are, however, widely used in the clinical setting. They have a high informative value, but also some limitations such as a ceiling effect. Pertinent methods regularly used in a clinical setting include but are not limited to the Berg Balance Scale, or the Tinetti Balance and Gait Test. These tests could be further specified, and their reliability and validity could be further increased by utilizing and augmenting objective data from an exoskeleton. In our review, we only detected one original study [62] using exteroceptive sensors to assess motor performance in humans. However, it is conceivable that such sensors can potentially be used to measure certain aspects of motor performance or, through sensor fusion, can improve the results of other measurements. For the test procedure itself, sufficient familiarization of the participants and minimal disturbance by the exoskeleton must be ensured. Given that the number of persons over 60 years of age will double to more than two billion within the next 30 years, and in light of a decreased mobility of older adults due to sarcopenia and other limitations or medical conditions, exoskeletons may be used to not only maintain mobility, but also to detect and train weaknesses with regard to motor performance through new sensor technology, control algorithm, design, and lightweight materials [109]. In the future, various target groups for lower-limb exoskeletons, including but not limited to older persons with and without cognitive and/or physical impairments, should be considered in research studies.

## 5. Conclusions

The results of our review show that particularly stationary and single-joint exoskeletons can be used to measure a wide range of motor performance parameters (RoM, strength, proprioception, gait parameters, and muscle stiffness) through built-in sensors. Most studies utilized exoskeletons with a (quasi-) anthropomorphic design to simplify the calculations. Regarding assessing certain motor performance parameters, for example, proprioception or spasticity, exoskeletons are considered more objective and specific than manual test procedures. However, since these parameters are usually only estimated from built-in sensor data, the quality and specificity of an exoskeleton to assess certain motor performance parameters must be ensured before an exoskeleton should be used in a research or clinical setting. To this end, comprehensive evaluation studies that examine validity, reliability, and accuracy of exoskeletons assessing motor performance parameters are still scarce. Furthermore, it must be noted that an exoskeleton itself may have an impact on motor performance, for example, due to the unfamiliar situation for the user or irritations caused by micro misalignments.

## Figures and Tables

**Table 2:** Overview and results of validity and reliability studies (Abbreviations: 6MWT, 6-min walk test; BM, baseline model; CP, cerebral palsy; DEP, dependent-movement type is known; FSR, force sensing resistor; ICC, intraclass correlation; IMU, inertial measurement unit; IND, independent movement type is not known; (i)SCI, (incomplete) spinal cord injury; n.d., not described; NN, feedforward neural network; MAE, mean absolute error; PGE, pantographic exoskeleton sensor RMSE, root mean square error; XGBoost, gradient boosting algorithm).

Author	Exoskeleton	Participants	Validation Tool	Protocol	Results
Joint Angles					
Hu et al. [38]	Lower extremity exoskeleton (full limb, stationary)	**N = 1** (1♂) **health:** all healthy	Vicon motion capture system (Oxford Metric, Oxford, UK)	Sit to stand exercise	**Human-robot hip angle deviation** - mean: 4.93° ± 3.05 (PGE) - mean: 4.94° ± 3.2 (hip encoder) **Human-robot knee angle deviation** - mean: 3.00° ± 1.64 (PGE) **Human-robot ankle angle deviation** - mean: 1.02° ± 0.79 (PGE)
Veneman et al. [42]	LOPES Exoskeleton (hip and knee; stationary)	**N = 10** **age:** 26 **health:** all unimpaired	PTI-VZ4000 mocap system from PhoeniX Technologies (Campbell, CA, USA)	Treadmill walking	- Only small deviations between the robotic and the motion capturing assessment - Not precise enough for inverse dynamics calculations
Koginov et al. [44]	Myosuit (hip and knee, mobile)	**N = 8** (4♂, 4♀) **health:** all healthy	Vicon motion capture system (Oxford Metric, Oxford, UK)	Standing and Treadmill walking with different support modes (1-3) and different speed (0.8 m/s, 1.3 m/s)	**Human-robot hip angle deviation** RMSE: - overall 2.5° ± 1 - error is bigger with more support and higher speed
d’Elia et al. [47]	Active pelvis orthosis (APO) (hip; mobile)	**N = 5** **age:** 29.2 ± 6.3 **health:** all healthy	optoelectronic system (SmartD, BTS, Milan, Italy)	Treadmill walking with three different speeds (slow, normal and fast; depending on leg length) and different modes (transparent, low, moderate and high assistance)	**Human-robot hip angle deviation** RMSE: - Transparent mode: 2.7° ± 0.8 (slow)–3.6° ± 1.2 (fast) - Low assistance: 4.0° ± 0.9 (slow)–5.2° ± 1.1 (fast) - Moderate assistance: 4.9° ± 0.7 (slow)–5.9° ± 1.0 (fast) - High assistance: 5.3° ± 0.7 (slow)–7.2° ± 1.1 (fast)
Park et al. [52]	Active Soft Orthotic Device (ankle; mobile)	n.d.	Goniometer	Moving the ankle freely in plantar and dorsiflexion	Mean error: - 0.135° ± 2.85 (IMU) - /0.255° ± 1.63 (strain sensor)
**Proprioception**					
Chisholm et al. [56]	Lokomat (full leg; stationary)	**N = 34** (26♂, 8♀) **age:** 39.5 ± 10.2 (abled bodied) 39.5 ± 9.7 (SCI) **health:** n = 17 abled bodied n = 17 SCI	Manual assessment	2 assessments of robotic lower limb joint proprioception separated by one week; manual assessment of proprioception	**Test–retest reliability** Hip: - ICC = 0.88 left, ICC = 0.94 right (control) - ICC = 0.97 left, ICC = 0.96 right (SCI) Knee: - ICC = 0.90 left, ICC = 0.91 right (control) - ICC = 0.95 left, ICC = 0.96 right (SCI) **Validity** - Movement detection score increases with increasing speed **Comparison to manual assessment** - Robotic assessment is more sensitive - No ceiling effect in robotic assessment
Domingo and Lam [58]	Lokomat (full leg; stationary)	**N = 46** (28♂, 18♀) **age:** 37.8 ± 14.1 (abled bodied) 40.5 ± 14.0 (SCI) **health:** n = 23 abled bodied n = 23 SCI	Manual assessment	First participant was moved to the target position for 5 s and second to the starting position passively; participant must replicate the target position; manual assessment of proprioception	**Test–retest reliability** Hip: - ICC = 0.493 (healthy) - ICC = 0.55 (iSCI) Knee: - ICC = 0.656 (healthy) - ICC = 0.882 (iSCI) **Comparison to manual assessment** - Both scores corelate
Dambreville et al. [55]	Electrohydraulic robotized ankle–foot orthosis (ankle; mobile)	**N = 25** (13♂, 12♀) **age:** 22.88 ± 2.63 **health:** all healthy	Only reliability study	Treadmill walking; exoskeleton induces perturbations during gait; push a button when they felt a perturbation	- ICC = 0.78 (95% CI: 0.45–0.91)
**Gait Phase, Spatio-temporal Gait Parameters and Walking Ability**					
Lonini et al. [60]	ReWalk (full leg; mobile)	**N = 11** (6♂, 5♀) **age:** 26.9 ± 14 **health:** n = 6 abled bodied n = 5 SCI	Number of steps (accelerometer)	Two 6MWT (1 min pause between) on a 30 m walkway	- Proposed score has a higher discriminatory power to distinguish between expert and non-expert users than just the number of steps
Kang et al. [64]	Powered hip exoskeleton (hip; mobile)	**Young** **n = 4** (3♂, 1♀) **age:** 23.5 ± 3.3 **Elderly** **n = 2** (2♀) **age:** 72.5 **health:** all healthy	Treadmill	Treadmill walking (different speed)	**Walking speed (RMSE)** - 0.094 m/s ± 0.043 (young) - 0.061 m/s (elderly)
Li et al. [62]	Unilateral rehabilitation exoskeleton robot (full leg; mobile)	**N = 10** (8♂, 2♀) **age**: 25 ± 4 **health:** all healthy	Vicon motion capture system (Oxford Metric, Oxford, UK)	- 5 m Level ground walking at self-selected speed (6 trials) - Treadmill walking (2/4/6 km/h) for 1 min	**Gait phase estimation (MAE in ms)** **(self-selected speed; 2 km/h; 4 km/h; 6 km/h)** - Heel strike: 20.5; 27.3; 24.4; 22.6 - Foot flat: 24.8; 25.8; 29.1; 34.0 - Heel off: 17.1; 28.2; 32.8; 31.0 - Toe off: −25.8; −31.1; −28.3; −30.7 - Back Swing highest: 14.1; 17.9; 19.7; 22.5 - Front swing lowest: 15.1; 16.5; 20.9; 19.5
Xia et al. [63]	Passive lower limb weight-bearing exoskeleton (full leg; mobile)	**N = 7** (6♂, 1♀) **age:** 25–30 **health:** all healthy	Image acquisition system (manual labelling)	- Treadmill walking (2, 4, and 6 km/h)	**Gait phase estimation accuracy (correct classified data points/total data points))** - average: 92.989% - left foot lift, right foot hang: 69% - left foot lift, right foot support: 96% - left foot hang, right foot lift: 82% - left foot hang, right foot support: 97% - left foot support, right foot lift: 94% - left foot support, right foot hang: 98% - left foot support, right foot support: 81%
Zhang et al. [45]	Single-joint robotic hip exoskeleton (hip; mobile)	**N = 7** (5♂, 2♀) **age:** 25.9 ± 3.8 **health:** all healthy	FSR Sensors in foot insole (offline)	- Treadmill walking (increasing speed) - Free walking (self-selected speed)	**Gait phase estimation (RMSE)** - 5.15% ± 1.82 (treadmill) - 5.53% ± 2.09 (free walking)
Crea et al. [68]	Active pelvis orthosis (APO) (hip; mobile)	**N = 7** (4♂, 3♀) **age:** 28.6 ± 4.9 **health:** all healthy	Sensor insoles	Treadmill walking with fast and slow speed in two modes (assistive and transparent)	**Gait phase estimation (RMSE)** - 0.26 rad (transparent mode) - 0.27 rad (assistive mode)
Yu et al. [70]	Portable knee exoskeleton (knee; mobile)	**N = 3** **age:** 25.3 ± 0.94	Foot switches	Walking on a treadmill and stair walking (ascending + descending) at steady and varying speed	**Gait phase estimation (RMSE)** Steady speed: - Walking (ANN): 4.10% - Walking (event based): 1.66% - Stairs ascending (ANN): 4.58% - Stairs ascending (event based): 4.55% - Stairs descending (ANN): 5.32% - Stairs descending (event based): 3.51% Varying speed: - Walking (ANN): 4.18% - Walking (event based): 11.0% - Stairs ascending (ANN): 6.66% - Stairs ascending (event based): 15.3% - Stairs descending (ANN): 8.03% - Stairs descending (event based): 18.5%
**Joint Torques and Strength**					
Cherni et al. [73]	Lokomat (full leg; stationary)	**N = 17** (9♂, 8♀) **age:** 10.0 ± 3.2 **health:** all CP	Handheld dynamometer	Isometric force measurement fixed joints angles (30° hip flexion, 45° knee flexion); producing and holding maximum strength for 5 s, each muscle group (hip flexors/extensors and knee flexors/extensors) measured separately	**Test–retest reliability** Inter-tester (single measurement): - ICC = 0.8 (hip flexion)–0.87 (hip extension) Inter-tester (average measurement): - ICC = 0.89 (hip flexion)–0.93 (hip extension) Intra-tester (single measurement): - ICC = 0.7 (knee extension)–0.87 (hip flexion) Intra-tester (average measurement): - ICC = 0.83 (knee extension)–0.93 (hip flexion) **Comparison robotic vs. manual assessment** - Strong correlation hip and knee flexors (0.76 and 0.60) - Moderate correlation for hip and knee extensors (0.53 and 0.52)
Galen et al. [71]	Lokomat (full leg; stationary)	**N = 18** (14♂, 4♀) **age:** 49.3 ± 11 **health:** all iSCI	Standard neurological classification of spinal cord injury (ASIA) scoring system	Isometric force fixed joints angles; producing and holding maximum strength for 5 s, muscle groups: hip flexors/extensors and knee flexors/extensors	- Non-linear relation between generated peak torque and ASIA Score
Bolliger et al. [74]	Lokomat (full leg; stationary)	N = 30 (8♂, 32♀) age: 25.7 ± 3.8 (healthy) 53.5 ± 16.5 (neurological disorders) health: n = 16 healthy n = 14 neurological disorders	Only reliability study	Isometric force measurement fixed joints angles (30° hip flexion, 45° knee flexion); producing and holding maximum strength for 5 s, each muscle group (hip flexors/extensors and knee flexors/extensors) measured separately	**Healthy** Inter-tester reliability - Single measurement: ICC = 0.72 (hip extension, left) – 0.95 (hip extension, right) - Average measurement: ICC = 0.91 (hip extension, left)–0.97 (knee extension, left; hip flexion, left; knee flexion, right) Intra-tester reliability - Single measurement: ICC = 0.71 (knee extension, right)–0.9 (hip extension, right) - Average measurement: ICC = 0.74 (hip extension, left)–0.9 (knee extension, right) **Neurological disorders** Inter-tester reliability - Single measurement: ICC = 0.66 (hip extension, less affected side)–0.97 (knee flexion, more affected side) - Average measurement: ICC = 0.85 (knee extension, more affected side)–0.96 (hip flexion, less affected side; knee flexion, more affected side) Intra-tester reliability - Single measurement: ICC = 0.5 (hip flexion, more affected side)–0.91 (knee flexion, less affected side) - Average measurement: ICC = 0.79 (hip flexion, more affected side)–0.96 (knee flexion, less affected side)
Rea et al. [76]	X1 exoskeleton (full leg; mobile)	**N = 8**	Biodex system; dynamometer	n.d.	- Test–retest repeatability was comparable to Biodex system
Molinaro et al. [46]	Robotic hip exoskeleton (hip; mobile)	**N = 5** **age:** 23.0 ± 2.1 **health:** all healthy	Vicon motion capture system (Oxford Metric, Oxford, UK) + Bertec force plates (Bertec, Columbus, OH, USA) + OpenSim	Walking on a treadmill Level ground Ramp ascent Ramp descent	**RMSE of estimated hip torque compared to ground truth:** Level ground: - 0.149 (Baseline model (BM) + known movement (DEP)) - 0.186 (BM + unknown movement type (IND)) - 0.071 (gradient boosting algorithm (XGBoost) + IND) - 0.097 (feedforward neural network (NN) + IND) Ramp ascent: - 0.188 (BM + DEP) - 0.580 (BM+ IND) - 0.092 (XGBoost + IND) - 0.127 (NN + IND) Ramp ascent: - 0.153 (BM + DEP) - 0.488 (BM + IND) - 0.082 (XGBoost + IND) - 0.083 (NN + IND)
**Stiffness/Spasticity/Impedance**					
Lunenburger et al. [36]	Lokomat (full leg; stationary)	**N = 42** **health:** all with neurological disorders	Modified Ashworth score	Automated movement of the tested joints; participant‘s legs are 100% unloaded	- Measured stiffness correlated with the Modified Ashworth score
Cherni et al. [79]	Lokomat Pediatric version (full leg; stationary)	**N = 16** (9♂, 7♀) **age:** 20 ± 3 **health:** all CP	Only reliability study	Lokomat L-STIFF Tool; Exoskeleton displace each joint with three different velocities (slow/medium/fast)	**Test–retest reliability** Intra-tester (same day): - ICC = 0.69 (fast knee extension)–0.95 (fast hip extension/medium hip flexion) Intra-tester (between days): - ICC = 0.49 (slow hip extension)–0.89 (medium knee extension/slow knee flexion) Inter tester: - ICC = 0.32 (slow hip extension)–0.7 (fast hip extension)

**Table 3:** Results of the quality assessment of validity and reliability studies. The reader is referred to National Heart, Lung and Blood Institute (2021) [35] for further details on the different items of the quality assessment tool (na, not applicable).

Author/Item	1	2	3	4	5	6	7	8	9	10	11	12	Sum
Bolliger et al. [74]	1	1	1	na	1	1	1	na	na	1	1	na	8
Cherni et al. [73]	1	1	1	na	1	1	1	na	na	1	1	na	8
Cherni et al. [79]	1	1	1	na	1	1	1	na	na	1	1	na	8
Chisholm et al. [56]	1	1	1	na	1	1	1	na	na	1	0	na	7
Crea et al. [68]	1	1	0	na	0	1	1	na	na	1	0	na	5
Dambreville et al. [55]	1	1	1	na	1	1	1	na	na	1	1	na	8
d’Elia et al. [47]	1	1	1	na	0	1	1	na	na	1	0	na	6
Domingo and Lam [58]	1	1	1	na	1	1	1	na	na	1	0	na	7
Galen et al. [71]	1	1	1	na	1	1	1	na	na	1	1	na	8
Hu et al. [38]	1	0	0	na	0	1	1	na	na	0	0	na	3
Kang et al. [64]	1	1	1	na	0	1	1	na	na	0	0	na	5
Koginov et al. [44]	1	1	0	na	1	1	1	na	na	1	1	na	7
Li et al. [62]	1	0	0	na	1	1	1	na	na	1	1	na	6
Lonini et al. [60]	1	1	1	na	0	1	1	na	na	1	0	na	6
Lunenburger et al. [36]	1	0	0	na	1	1	1	na	na	0	0	na	4
Molinaro et al. [46]	1	1	1	na	0	1	1	na	na	1	0	na	6
Park et al. [52]	1	0	0	na	0	1	1	na	na	0	0	na	3
Rea et al. [76]	0	0	0	na	0	0	0	na	na	0	0	na	0
Veneman et al. [42]	1	0	0	na	0	1	1	na	na	0	0	na	3
Xia et al. [63]	0	0	0	na	0	1	1	na	na	1	0	na	3
Yu et al. [70]	1	0	0	na	0	1	1	na	na	0	1	na	4
Zhang et al. [45]	1	1	0	na	0	1	1	na	na	0	0	na	4

## Data Availability

Not applicable.
